# A Screen for Spore Wall Permeability Mutants Identifies a Secreted Protease Required for Proper Spore Wall Assembly

**DOI:** 10.1371/journal.pone.0007184

**Published:** 2009-09-25

**Authors:** Yasuyuki Suda, Rachael K. Rodriguez, Alison E. Coluccio, Aaron M. Neiman

**Affiliations:** Department of Biochemistry and Cell Biology, Stony Brook University, Stony Brook, New York, United States of America; Universidade de Sao Paulo, Brazil

## Abstract

The ascospores of *Saccharomyces cerevisiae* are surrounded by a complex wall that protects the spores from environmental stresses. The outermost layer of the spore wall is composed of a polymer that contains the cross-linked amino acid dityrosine. This dityrosine layer is important for stress resistance of the spore. This work reports that the dityrosine layer acts as a barrier blocking the diffusion of soluble proteins out of the spore wall into the cytoplasm of the ascus. Diffusion of a fluorescent protein out of the spore wall was used as an assay to screen for mutants affecting spore wall permeability. One of the genes identified in this screen, *OSW3* (*RRT12*/*YCR045c*), encodes a subtilisin-family protease localized to the spore wall. Mutation of the active site serine of Osw3 results in spores with permeable walls, indicating that the catalytic activity of Osw3 is necessary for proper construction of the dityrosine layer. These results indicate that dityrosine promotes stress resistance by acting as a protective shell around the spore. *OSW3* and other *OSW* genes identified in this screen are strong candidates to encode enzymes involved in assembly of this protective dityrosine coat.

## Introduction

In response to starvation, specifically the absence of a nitrogen source in the presence of a non-fermentable carbon source, *MATa*/*MATα* diploid cells of the yeast *Saccharomyces cerevisiae* differentiate to form haploid spores (for review see [1]). Spores are quiescent, stress-resistant cells that can persist until nutrients are re-introduced into the environment. In particular, spores may be adapted to survive passage through the gut of insects as a means of dispersing in the environment [2].

Central to the ability of spores to survive is a thick coat, or spore wall, that surrounds each spore [3]. This wall provides the spore with increased resistance to a wide variety of environmental conditions including heat, high osmolarity, acidic and basic pH, degradative enzymes, and ether vapor [2]–[4]. The spore wall is more extensive than the vegetative cell wall and is constructed of four different layers. The inner two layers, composed of mannans and β-glucans, are similar in composition to the vegetative cell wall [5]. The two outer layers are unique to the spore. First, there is a layer composed of chitosan, a glucosamine polymer formed by deacetylation of chitin [6]–[8]. Surrounding the chitosan, is a layer consisting primarily of the cross-linked amino acid dityrosine [9]. The chitosan appears to be the substrate on which the dityrosine layer is formed. Mutation of the *DIT1* gene, required for synthesis of a dityrosine precursor, results in the loss of a dityrosine layer but leaves an intact chitosan layer, while mutation of the chitin synthase *CHS3*, eliminates both the chitosan and dityrosine layers while leaving the inner layers of the spore wall intact [4], [8]. Mutants lacking either the dityrosine layer or both the chitosan and dityrosine layers form spores that are stress-sensitive, indicating that these layers are primarily responsible for the spores' increased resistance to environmental insults [4], [8].

A screen for mutants that form ether-sensitive spores identified a number of genes involved in spore wall assembly [10]. Characterization of the mutants revealed that the spore wall is assembled from inside to out, beginning with the mannan layer and ending with the formation of the dityrosine layer. While many of the mutants displayed clear defects in cytological or biochemical analyses of the spores, some of the mutants formed spores with walls that were indistinguishable from wild type by fluorescence or electron microscopy but nonetheless displayed increased susceptibility to environmental challenge [10]. This class of mutants is presumably defective in more subtle aspects of wall assembly and highlights the need for finer assays to detect defects in spore wall structure.

Subtilisin-family proteases encompass a large number of structurally related serine proteases within which are several sub-groups including the bacterial subtilisin, proprotein convertase (or kexin), and proteinase K families [11]. Most, if not all of the eukaryotic subtilisin-related proteases are either secreted or function within the secretory pathway. In *S. cerevisiae*, there are four subtilisin family members, Kex2, Prb1, Ysp3, and Rrt12/Ycr045c. Kex2 is localized to the trans-Golgi and is involved in processing of alpha factor and other proproteins and Prb1 is a vacuolar protease [12]–[14]. The function and localization of Ysp3 and Rrt12/Ycr045c have not been reported, though Ysp3 is likely a paralog of Prb1 [15]. A phylogenetic analysis of the subtilisin superfamily places Kex2 within the kexin subfamily, while the remaining yeast proteins are in the proteinase K branch of the subtilisins [11].

We used a secreted form of green fluorescent protein (GFP) to investigate the permeability of the spore wall to protein-sized molecules. We report that GFP is retained within the wild-type spore wall but leaks out of the wall in mutants lacking the dityrosine layer. Leakage of GFP from the spore wall was used as an assay to screen a collection of mutants in sporulation-induced genes. Several novel genes in which mutants display increased spore wall permeability were identified. One of the genes, *RRT12*/*YCR045c*, which we propose to name *OSW3* (for Outer Spore Wall), encodes a sporulation-specific, secreted protease of the subtilisin family. Mutation of the presumptive catalytic serine in Osw3 blocks the processing of Osw3 pro-domain and results in increased spore wall permeability, suggesting that proteolytic activity of Osw3 is necessary for proper assembly of the outer layers of the spore wall.

## Methods

### Yeast Strains and Media

Unless otherwise noted, standard methods and media were used [16]. The strains used in this study are listed in Table 1. All strains in this study are in the fast-sporulating SK-1 strain background [17]. Gene disruption and insertion were done using PCR- generated DNA cassettes [18]. Strain YS28 was generated using MJY5 and MJY6 as primers and pFA6a-HIS3MX6 to generate knockouts of *FKS3* in AN117-4B and AN117-16D followed by mating of the resulting haploids. Deletion of *OSW3* was performed in the same way using YSO148 and YSO149 as primers and pFA6a-kanMX6 in AN117-4B and AN117-16D to create YS414 and YS415, respectively. YS416 was generated by the mating of these haploids. C-terminal GFP tagging of *OSW3* was performed using YSO135 and YSO136 as primers and pFA6a-GFP-HIS3MX6 as a template to create YS305. YS308 was generated in the same way as YS305 using the same primers and pFA6a-3HA-kanMX6 as template. Bsu36I digested pRS304-OSW3-HA and pRS304-OSW3-SA-3HA were integrated into *TRP1* locus of YS414 and YS415, followed by mating of resulting haploids to create YS478 and YS479, respectively.

**Table 1:** *S. cerevisiae* strains used in this study.

Strain	Genotype	Source
AN117-4B	*MAT*α *ura3 leu2 trp1 his3Δsk arg4-NspI lys2 ho::LYS2 rme1::LEU2*	[39]
AN117-16D	*MAT* **a** *ura3 leu2 trp1 his3Δsk lys2 ho::LYS2*	[39]
AN120	*MAT* **a** */MAT*α *ARG4/arg4-NspI his3ΔSK/his3ΔSK ho::LYS2/ho::LYS2 leu2/leu2 lys2/lys2 RME1/rme1::LEU2 trp1::hisG/trp1::hisG ura3/ura3*	[39]
AN262	*MAT* **a** */MAT*α *ARG4/arg4-NspI his3ΔSK/his3ΔSK ho::LYS2/ho::LYS2 leu2/leu2 lys2/lys2 RME1/rme1::LEU2 trp1::hisG/trp1::hisG ura3/ura3 chs3Δ::his5^+^/chs3Δ::his5^+^*	[40]
AN264	*MAT* **a** */MAT*α *ARG4/arg4-NspI his3ΔSK/his3ΔSK ho::LYS2/ho::LYS2 leu2/leu2 lys2/lys2 RME1/rme1::LEU2 trp1::hisG/trp1::hisG ura3/ura3 dit1Δ::his5^+^/dit1Δ::his5^+^*	[40]
YS28	*MAT* **a** */MAT*α *ARG4/arg4-NspI his3ΔSK/his3ΔSK ho::LYS2/ho::LYS2 leu2/leu2 lys2/lys2 RME1/rme1::LEU2 trp1::hisG/trp1::hisG ura3/ura3 fks3Δ::his5^+^/fks3Δ::his5^+^*	this study
ER309	*MAT* **a** */MAT*α *ARG4/arg4-NspI his3ΔSK/his3ΔSK ho::LYS2/ho::LYS2 leu2/leu2 lys2/lys2 RME1/rme1::LEU2 trp1::hisG/trp1::hisG ura3/ura3 gas2Δ::HIS3/gas2::HIS3 gas4Δ::kanMX2/gas4ΔkanMX2*	[23]
YS414	*MAT*α *ura3 leu2 trp1 his3Δsk arg4-NspI lys2 ho::LYS2 rme1::LEU2 osw3Δ::kanMX6*	this study
YS415	*MAT* **a** *ura3 leu2 trp1 his3Δsk lys2 ho::LYS2 osw3Δ::kanMX6*	this study
YS416	*MAT* **a** */MAT*α *ARG4/arg4-NspI his3ΔSK/his3ΔSK ho::LYS2/ho::LYS2 leu2/leu2 lys2/lys2 RME1/rme1::LEU2 trp1::hisG/trp1::hisG ura3/ura3 osw3Δ::kanMX6/osw3Δ::kanMX6*	this study
YS305	*MAT*α *ura3 leu2 trp1 his3Δsk arg4-NspI lys2 ho::LYS2 rme1::LEU2 OSW3-GFP:: his5^+^*	this study
YS308	*MAT*α *ura3 leu2 trp1 his3Δsk arg4-NspI lys2 ho::LYS2 rme1::LEU2 OSW3-3HA::kanMX6*	this study
YS478	*MAT* **a** */MAT*α *ARG4/arg4-NspI his3ΔSK/his3ΔSK ho::LYS2/ho::LYS2 leu2/leu2 lys2/lys2 RME1/rme1::LEU2 trp1::hisG::TRP1-OSW3-3HA/trp1::hisG::TRP1-OSW3-3HA ura3/ura3 osw3Δ::kanMX6/osw3Δ::kanMX6*	this study
YS479	*MAT* **a** */MAT*α *ARG4/arg4-NspI his3ΔSK/his3ΔSK ho::LYS2/ho::LYS2 leu2/leu2 lys2/lys2 RME1/rme1::LEU2 trp1::hisG::TRP1-osw3-S365A-3HA/trp1::hisG::TRP1-osw3-S365A-HA ura3/ura3 osw3Δ::kanMX6/osw3Δ::kanMX6*	this study
K8409	*MAT* ***a***/*MATα his3*/*his3 HO*/*HO LEU2::pURA3-tetR-GFP*/*LEU2::pURA3-tetR-GFP lys2*/*lys2 REC8::HA3-URA3*/*REC8::HA3-URA3 trp1*/*trp1 URA3::tetO_224_*/*URA3::tetO_224_*	[25]
MYA-1844	as K8409, plus *gis1Δ::HIS3MX6*/*gis1Δ::HIS3MX6*	[25]
MYA-2058	as K8409, plus *osw1Δ::HIS3MX6*/*osw1Δ::HIS3MX6*	[25]
MYA-1983	as K8409, plus *osw2Δ::HIS3MX6*/*osw2Δ::HIS3MX6*	[25]
MYA-1824	as K8409, plus *osw3Δ::HIS3MX6*/*osw3Δ::HIS3MX6*	[25]
MYA-1946	as K8409, plus *osw4Δ::HIS3MX6*/*osw4Δ::HIS3MX6*	[25]
MYA-2022	as K8409, plus *osw5Δ::HIS3MX6*/*osw5Δ::HIS3MX6*	[25]
MYA-1815	as K8409, plus *pbp2Δ::HIS3MX6*/*pbp2Δ::HIS3MX6*	[25]
MYA-1857	as K8409, plus *ydr326cΔ::HIS3MX6*/*ydr326cΔ::HIS3MX6*	[25]
MYA-2068	as K8409, plus *ypl033cΔ::HIS3MX6*/*ypl033cΔ::HIS3MX6*	[25]
MYA-2078	as K8409, plus *ypl200wΔ::HIS3MX6*/*ypl200wΔ::HIS3MX6*	[25]
MYA-2087	as K8409, plus *ydr263cΔ::HIS3MX6*/*ydr263cΔ::HIS3MX6*	[25]
MYA-1876	as K8409, plus *mam1Δ::HIS3MX6*/*mam1Δ::HIS3MX6*	[25]
MYA-1835	as K8409, plus *ydl186wΔ::HIS3MX6*/*ydl186wΔ::HIS3MX6*	[25]
MYA-1994	as K8409, plus *far10Δ::HIS3MX6*/*far10Δ::HIS3MX6*	[25]

### Plasmids

The oligonucleotides and plasmids used in this study are listed in Tables 2 and 3, respectively. pRS424-SPR1-GFP was constructed by PCR amplification of the *SPR1* gene using ANO122 and ANO123 from genomic DNA. This PCR product carries 540 bp of the *SPR1* upstream region as well the coding region but lacks a stop codon. This fragment was digested with *Sac*I and *Not*I and cloned into similarly digested pRS424 [19] to create pRS424-SPR1. The coding region of GFP was then cloned out of pGFP (a gift of J. Leatherwood) as a *Not*I-*Bam*HI fragment and cloned into the similarly digested pRS424-SPR1. To generate pRS424-ssGFP, the promoter and first 22 codons of *SPR1* were amplified from pRS424-SPR1-GFP using the ANO122 and SPR1-Trunc primers. The resulting product was digested with *Sac*I and *Not*I and cloned into the backbone of similarly digested pRS424-SPR1-GFP. To create pRS304-OSW3-3HA, the *OSW3-3HA* coding region and 600 bp of the promoter were amplified using genomic DNA of YS308 as a template and YSO139 and HT66 as primers. The resulting PCR product was digested with *Xba*I and *Bgl*II, and cloned into *Spe*I-*Bam*HI digested in pRS304. pRS304-osw3-S365A-3HA was generated using a site directed mutagenesis kit (Stratagene, La Jolla, CA) and oligos YSO141 and YSO142. To generate pRS424-OSW3-3HA and pRS424-osw3-S365A-3HA, *Sac*1-*Kpn*1 fragments from pRS304-OSW3-3HA and pRS304-OSW4-S365A-3HA were cloned into similarly digested pRS424. pRS424-P_TEF2_-OSW3-3HA was made as follows. *OSW3-3HA* was amplified from pRS304-OSW3-3HA using YSO140 and HT66. The resulting PCR product was digested with *Spe*I and *Bgl*II, and cloned into *Spe*I-*Bam*HI digested pRS424- P_TEF2_. To create pRS424- P_TEF2_-OSW3-S365A-3HA, a *Bam*HI and *Apa*I fragment from pRS304-osw3-S365A-3HA containing *osw3-S365A-3HA* was cloned into similarly digested pRS424- P_TEF2_-OSW3-3HA. To generate pRS424-OSW3-GFP, PCR was performed using genomic DNA from YS305 as template, and YSO139 and HT66 as primers. This PCR product was digested with *Xba*I and *Bgl*II and cloned into *Spe*I-*Bam*HI digested pRS424.

**Table 2:** Primers used in this study.

Name	Sequence (5′-3′)
ANO122	GTTCGGATCCGAGCTCAAAGGGCCAAGAAGTAAGGC
ANO123	GTTCGGATCCGCGGCCGCAATGACATTGGTTAGGATATTTCC
SPR1-Trunc	GTTCTTGCGGCCGCATATGGAAACAGGATTACAG
MJY5	GCATATTGGGAAAAGAAAAACATACTGAGAAGGAAAGTTAAGGAGTTGAATCGGATCCCCGGGTTAATTAA
MJY6	AGGCACCAGCTCGCTTTATCAGCTACTCAAAATAACTTTTTTTTTTTATCCGAATTCGAGCTCGTTTAAAC
YSO148	GTCTAAGAAGAAAGAAATAG
YSO149	CCACAGCTAACCTCATATTT
YSO135	TACCTCTTGGTGAGATTCGATTGAAGAGGCGTGATTTTATGAAAAATTTGCGGATCCCCGGGTTAATTAA
YSO136	TGAAAAATGAAAAGTACAAGAATACGCAAGAGAAGAGCGAAAAAACTAATGAATTCGAGCTCGTTTAAAC
YSO139	GGAGGATCTAGAACGCCTCGTTGCTGTTGCGG
HT66	GAAGAATTCAGATCTATATTACCCTGTTATCC
YSO141	GTCAGGTACCGCTATGTCGACGCCCATTG
YSO142	CAATGGGCGTCGACATAGCGGTACCTGAC
YSO220	TCCAAGAACTTAGTGAACGCGCTGGCGGCAAGCCCACTAGTGGCTGATATTGTGC
YSO221	GCACAATATCAGCCACTAGTGGGCTTGCCGCCAGCGCGTTCACTAAGTTCTTGGA
YSO224	GCAATTGAGGCGGCATTCTCCATCGGTTCCTTCCGCGGCGTGACCAT
YSO225	GTTGTCGATGAACTGTCTCCATGACCTGTT

**Table 3:** Plasmids used in this study.

Name	Description	Source
pRS424-SPR1-GFP	*TRP1*, 2 µ, *SPR1-GFP*	this study
B1913 (SPR1-GFP-URA3)	*URA3*, 2 µ, *SPR1-GFP*	Joanne Engebrecht
pRS424-ssGFP	*TRP1*, 2 µ, *SPR1 signal sequence-GFP*	this study
pRS424-OSW3-GFP	*TRP1*, 2 µ, *OSW3-GFP*	this study
pRS304-OSW3-3HA	*TRP1*, integration, *OSW3-3HA*	this study
pRS304-osw3-S365A-3HA	*TRP1*, integration, *osw3-S365A-3HA*	this study
pRS424-OSW3-3HA	*TRP1*, 2 µ, *OSW3-3HA*	this study
pRS424-osw3-S365A-3HA	*TRP1*, 2 µ, *osw3-S365A-3HA*	this study
pRS424- P_TEF2_-OSW3-3HA	*TRP1*, 2 µ, *OSW3-3HA*	this study
pRS424- P_TEF2_-osw3-S365A-3HA	*TRP1*, 2 µ, *osw3-S365A-3HA*	this study

### Leakage assays

Leakage of ssGFP and Spr1-GFP from the spore wall was assayed by transforming strains to be tested with high copy plasmids carrying *SPR1-GFP* or *ssGFP* and sporulating them in 2% KOAc at 30°C for 20 hr. The GFP signal was then examined by fluorescence microscopy and at least 200 individual asci were scored as to whether GFP was seen only in the spore wall or throughout the ascal cytoplasm.

### Microscopy

Fluorescence microscopy was performed using Zeiss Axioplan2 (Carl Zeiss, Thornwood, NY) microscope with a Zeiss mRM Axiocam and processed using Zeiss Axiovision 4.7 software.

### Immunoblotting

For the western blot analysis of Osw3, cells were transferred to sporulation medium and total protein was prepared using β-mercaptoethanol/NaOH extraction [20]. Proteins were separated by SDS-PAGE, and detected by Western Blot using an anti-HA monoclonal antibody (12CA5). Bands were visualized using HRP-conjugated sheep anti-mouse IgG antibody (GE Healthcare, Little Chalfont, United Kingdom).

### PNGase F digestion

PNGase F digestion was performed as follows. Protein samples prepared from 0.5 units (OD_600_) of the sporulating cells were diluted into 100 µl of 1× denaturing buffer (0.5% SDS, 40 mM DTT), boiled for 5 min and cooled to room temperature. 20 µl of 10×G7 buffer (0.5 M sodium phosphate, pH 7.5) and 20 µl of 10% NP-40 were added and the total volume was increased to 200 µl. The samples were then split into 2 tubes, 1 µl of PNGase F (500 units) (New England Biolabs, Beverly, MA) was added to one of the tubes, and both samples were incubated for 16 h at room temperature before SDS PAGE.

### Assays of dityrosine fluorescence

For the patch assay, cells were grown for 2 days on YPD plates, and then replica-plated onto nitrocellulose filters on SPO plates and incubated for 3 days at 30°C. The filters were then transferred to petri dishes containing 200 µl of H_2_O, 70 µl of zymolyase, and 15 µl of β-mercaptoethanol for 5 h at 30°C. The filters were then moved to dishes containing 0.1 M NaOH to give the basic pH required for the fluorescence of dityrosine, then exposed to UV (302 nm) and photographed.

For quantitation of dityrosine fluorescence, cells were sporulated on solid medium and then transferred to a microscope slide in a solution of 5% NH_4_OH to raise the pH. Images were collected at fixed exposure times using a filter set optimized for dityrosine fluorescence (Ex. 320 nm; Em 410 nm) (Omega Optical, Brattleboro, VT). To calculate fluorescence values, fluorescence intensity was measured at two points along the edge of a spore and at a point just outside the spore was measured as a background control. The background value was subtracted from the average of the values obtained from the spore periphery to generate a fluorescence level for each spore.

### β-glucanase resistance assays

Sensitivity to β-glucanase digestion was assessed using Zymolyase 100T (US Biologicals). Percent survival was assessed by titering the cultures before and after a one hour incubation with Zymolyase, as described previously [2].

## Results

### Soluble, secreted proteins are retained in the spore wall

**Figure 1:**
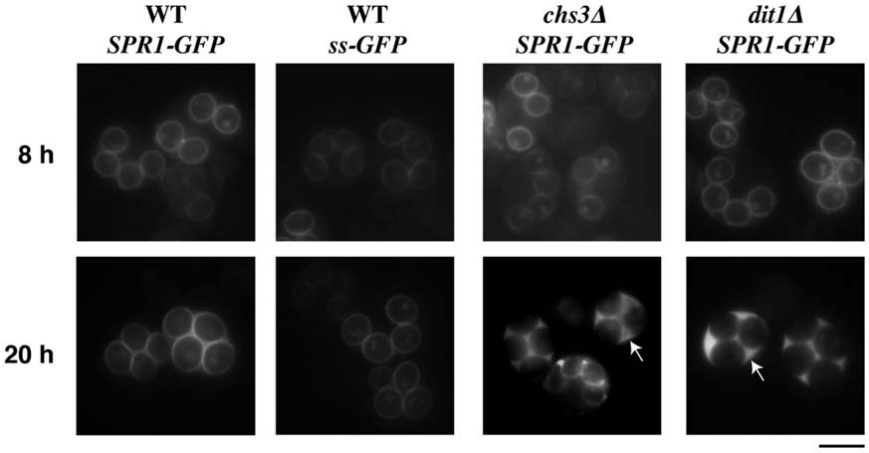
Spr1-GFP is a marker for spore wall permeability. AN120 (wild-type), AN262 (*chs3Δ*), and AN264 (*dit1Δ*) harboring high copy plasmids expressing *SPR1-GFP* or *ssGFP* (pRS424-SPR1-GFP or pRS424-ssGFP) were examined by fluorescence microscopy 8 hours or 20 hours after transfer to sporulation medium. Arrows indicate asci displaying fluorescence in the ascal cytoplasm. Bar = 5 µm.

*SPR1* encodes a sporulation specific exo-β-glucanase that is likely involved in assembly of the spore wall [21], [22]. To examine the localization of Spr1, an Spr1-GFP fusion protein was constructed. When expressed from the native *SPR1* promoter in wild-type cells, this fusion was found to localize to the forming prospore membrane in meiotic cells and to the spore wall of mature spores (Figure 1). Spr1-GFP fluorescence persisted as a tight ring even after many days of incubation, suggesting that the protein is tightly associated with the wall. Because Spr1 lacks consensus sequences for GPI anchor modification or other motifs associated with covalent linkage to the carbohydrate layers of the cell wall, this restricted localization suggests that there is some barrier to the diffusion of Spr1-GFP from the spore wall.

The Spr1-GFP fusion protein has a predicted molecular mass of ∼80 kD, not including possible glycosylation of the protein. Therefore, the spore wall could be permeable to relatively small molecules and still retain Spr1-GFP. To examine the ability of the wall to retain smaller proteins, the promoter and signal sequence of *SPR1* were fused directly to GFP. The resulting fusion, ssGFP, is also retained within the spore wall when expressed in wild-type cells (Figure 1). Thus, the spore wall prevents the diffusion of small proteins, at least down to the size of GFP (∼27 kD), from within the wall.

### The dityrosine layer is essential for this permeability barrier

**Figure 2:**
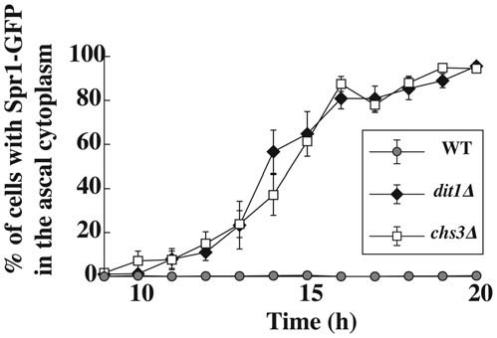
The dityrosine layer is essential for the Spr1-GFP diffusion barrier. AN120 (wild-type), AN262 (*chs3Δ*), and AN264 (*dit1Δ*) harboring a high copy plasmid expressing *SPR1-GFP* (pRS424-SPR1-GFP) were transferred to sporulation medium and, at the indicated time points after transfer, cells were collected and the percentage of asci displaying Spr1-GFP fluorescence throughout the ascus was determined. More than 200 cells were analyzed at each time point. Values shown are the averages of three independent experiments. The vertical lines indicate the range of the values.

To determine if the outer layers of the spore wall serve as a barrier to retain Spr1-GFP in the wall, a plasmid carrying the same Spr1-GFP fusion was transformed into a *chs3* mutant, which forms spores lacking both the chitosan and dityrosine layers, and a *dit1* mutant, which forms spores lacking only the dityrosine layer [4], [8]. Spr1-GFP behavior was similar in both strains. Specifically, during prospore membrane formation and at early time points after prospore membrane closure, GFP fluorescence was limited to the prospore membrane compartment. However, the GFP subsequently diffused out of the spore wall and was retained in the ascal cytoplasm so that by twenty hours after transfer to sporulation medium, 100% of the cells displayed fluorescence throughout the ascus (Figure 1). A time course analysis of the percentage of asci displaying Spr1-GFP fluorescence throughout the ascus suggested that the rate at which Spr1-GFP diffused out of the spore wall was comparable in both the *chs3* and *dit1* strains (Figure 2). Because the absence of the dityrosine layer alone leads to a similar rate of diffusion as the absence of both the chitosan and dityrosine layers, these data suggest that the dityrosine layer is the primary barrier to diffusion of Spr1-GFP from the wall.

### β-glucan defects result in uneven distribution within the wall

**Figure 3:**
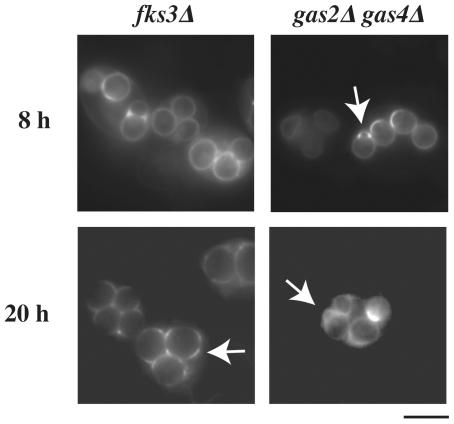
Mutants affecting the inner spore wall alter the distribution of Spr1-GFP. YS28 (*fks3Δ*) and ER309 (*gas2Δ gas4Δ*) harboring a high copy plasmid expressing *SPR1-GFP* (pRS424-SPR1-GFP) were examined by fluorescence microscopy 8 hours or 20 hours after transfer to sporulation medium. Arrows indicate abnormalities in the GFP fluorescence from the spore wall. Bar = 5 µm.

The effect of mutations that alter the inner layers of the spore wall on the localization of Spr1-GFP was also examined. Mutation of the *GAS2* and *GAS4* genes, encoding sporulation-specific β1,3 glucanosyltransferases, results in inner spore wall defects [23]. Though the outer chitosan and dityrosine layers are still present on the *gas2 gas4* spore, the connection between the outer and inner spore wall layers is disrupted at multiple points around the surface of the spore and disorganized wall materials are seen to accumulate at these sites [23]. To examine whether these disruptions alter the permeability of the spore wall, Spr1-GFP was introduced into a *gas2 gas4* strain. In *gas2 gas4* spores, Spr1-GFP was retained within the wall as in wild-type cells, but rather than being uniformly distributed throughout the wall it displayed a patchy distribution with discrete sites of concentration within the wall (Figure 3). These sites likely correspond to regions of discontinuity between the inner and outer spore wall layers. Similar results were obtained in *fks3* mutants, defective in a putative catalytic subunit of β1,3-glucan synthase [24](Figure 3). These results reveal that alteration of the inner layers of the spore wall can occur without altering the permeability of the outer spore wall to protein-size molecules. Diffusion of GFP proteins out of the spore wall can therefore be used as an assay to specifically identify mutants with outer spore wall defects.

### A screen for mutants with permeable spore walls

The ssGFP fusion was used to screen through a collection of ∼300 strains deleted for sporulation-induced genes [25]. This same collection has been previously screened for chromosome segregation defects, defects in the production of visible spores, and for ether sensitivity [10], [25]. Each strain was transformed with a plasmid expressing ssGFP from the *SPR1* promoter, sporulated, and asci from each strain were examined in the fluorescence microscope for localization of ssGFP after 24 hours on sporulation medium. Strains displaying four-spored asci with abnormal GFP fluorescence were retested and the percentage of asci displaying GFP fluorescence in the ascal cytoplasm was determined.

**Figure 4:**
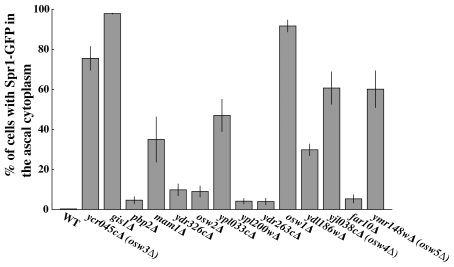
Leakage phenotypes of mutants found in the ssGFP screen. For each mutant identified in the visual screen, the distribution of Spr1-GFP was analyzed by fluorescent microscopy 20 hours after shift to sporulation medium. Data shown are the averages of three experiments. The vertical lines indicate the range of the values.

After this second round of screening, 14 mutants remained that reproducibly displayed an increased frequency of ascal GFP fluorescence. The mutants were next transformed with the Spr1-GFP plasmid to determine if they were similarly permeable to the larger fusion protein. All the mutants displayed comparable levels of leakage of both GFP proteins. The extent of this phenotype in all the mutants was quantitated using Spr1-GFP and was found to vary from 100% of the asci (*gis1Δ*) displaying fluorescence down to ∼5% (Figure 4).

Spore wall defects in three of the mutants, *osw1Δ, osw2Δ*, and *gis1Δ* have been characterized previously [10], [26]. *GIS1* encodes a transcription factor required for expression of the *DIT1* gene essential for dityrosine synthesis [10], [27]. *OSW1* encodes a spore wall localized protein required for assembly of both the chitosan and dityrosine layers [10], [26]. The nature of the spore wall defect in *osw2Δ* mutants is not understood, but it is also proposed to affect the outer layers of the spore wall [10].

**Figure 5:**
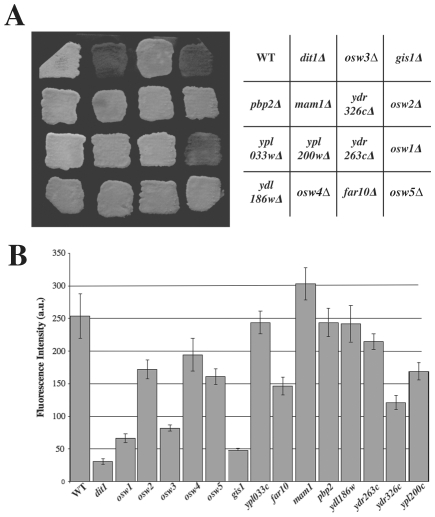
Dityrosine fluorescence in mutants identified in the visual screen. (A) Analysis of dityrosine fluorescence by patch assay. Each mutant was sporulated on a nitrocellulose filter and dityrosine fluorescence was visualized under UV light. AN120 (wild type) and AN264 (*dit1Δ*) were analyzed as positive and negative controls, respectively. (B) Quantitation of dityrosine fluorescence in each mutant strain. Each strain was sporulated and dityrosine fluorescence was quantified at the spore periphery by fluorescence microscopy. For each strain, fluorescence was measured in ten different spores. Mean values (in arbitrary units) are shown. Bars indicate standard error.

To examine if the remaining mutants affected the outer layers of the spore wall, dityrosine fluorescence of the spores was examined (Figure 5). By patch assay, only *osw1Δ* and *gis1Δ*, displayed an obvious loss of fluorescence comparable to a *dit1Δ* mutant (Figure 5A). To examine the mutants more quantitatively, we used fluorescence microscopy to quantify the dityrosine signal at the periphery of wild type and mutant spores. (Figure 5B). This analysis revealed that, in addition to *osw1Δ* and *gis1Δ*, the *rrt12*/*ycr045cΔ* mutant had strongly reduced dityrosine fluorescence. These three mutants also display the strongest leakage phenotypes (Figure 4), confirming the importance of the dityrosine layer in establishing the permeability barrier of the spore wall. Several other mutants display modestly reduced levels of dityrosine in their spore walls, consistent with the idea that these mutants have defects in organization of the outer spore wall layers.

Besides *osw1* and *gis1*, the mutants with the strongest leakage phenotypes are *rrt12*/*ycr045c*, *loh1*/*yjl038c* and *ymr148w*. Two of these genes, *RRT12*/*YCR045c* and *LOH1*/*YJL038c*, encode proteins with predicted signal peptides and the third, *YMR184w*, has a predicted N-terminal transmembrane domain. These sequence features suggest that these gene products may be secreted and therefore, directly involved in spore wall assembly. We designate these three genes *OSW3* (*YCR045c*), *OSW4* (*YJL038c*) and *OSW5* (*YMR148w*) for Outer Spore Wall.

### The *osw* mutants are sensitive to digestion by β-glucanases

**Figure 6:**
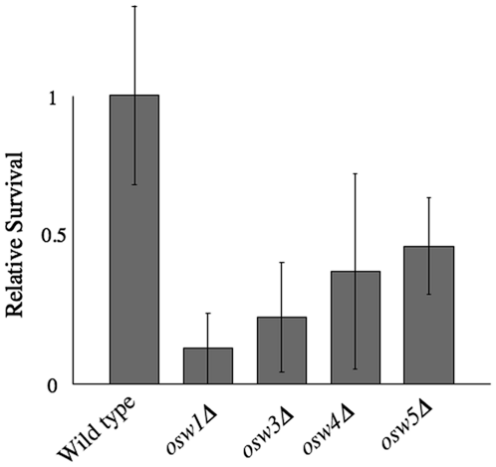
*osw* mutant spores are sensitive to digestion by β-glucanases. AN120 (wild type) and the indicated *osw* mutant strains were sporulated and the survival of the spores assessed after exposure to Zymolyase. Percent survival for each strain was normalized to the average survival of the wild type strain. Data shown are the averages of at least four experiments. The vertical lines indicate one standard deviation.

The leakage assay reveals that spore wall blocks the passage of protein size molecules from the periplasmic space out of the spore wall. The wall also likely presents a similar barrier to the entry of protein-sized molecules. Vegetative cells are killed by prolonged exposure to β-glucanase enzymes. These enzymes digest the β-1,3 glucans within the cell wall leading to loss of wall rigidity and cell lysis. Spores are resistant to β-glucanase digestion, probably because the β-glucan layer of the spore wall is shielded by the outer wall layers. Therefore, to examine if the *osw* mutations also increased the permeability of the wall to exogenous proteins, the resistance of *osw* mutant spores to β-glucanase digestion was examined (Figure 6). Wild type, *osw1Δ*, *osw3Δ*, *osw4Δ*, and *osw5Δ* mutant strains were sporulated and spores were titered both before and after one hour of treatment with β-glucanases. All four mutants were more sensitive to digestion than the wild-type spores with survival ranging from 12% (*osw1Δ*) to 47% (*osw5Δ*) that of wild type. These results indicate that the increased permeability of these mutants allows movement of proteins both into and out of the spore wall.

### *OSW3* encodes a subtilisin-related protease that is localized to the spore wall

**Figure 7:**
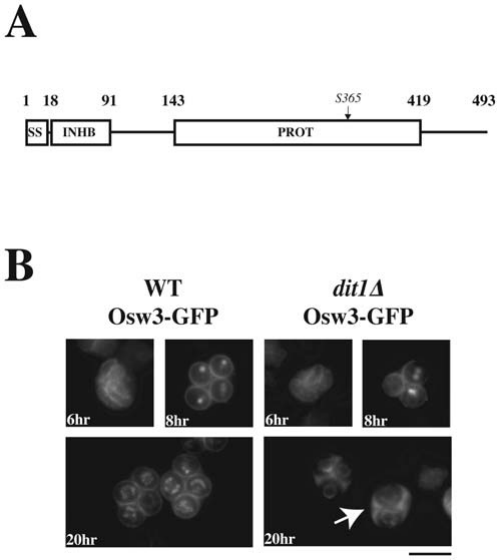
*OSW3* encodes a subtilisin-related protease that localizes to the spore wall. (A) Schematic of Osw3: SS = signal sequence; INHB = inhibitory pro-domain; PROT = protease catalytic domain. (B) AN120 (wild-type) and AN264 (*dit1Δ*) carrying a high copy OSW3-GFP plasmid (pRS424-OSW3-GFP) were analyzed by fluorescence microscopy at 5, 8, and 20 hours after transfer to sporulation medium. Arrow indicates fluorescence in the ascal cytoplasm of the *dit1Δ* mutant. Bar = 5 µm.

The predicted protein sequence of Osw3 includes an amino terminal signal peptide followed by regions of homology to subtilisin family proteases, including both a negative regulatory pro region and a carboxy terminal catalytic domain (Figure 7A). Two of the other subtilisin-family proteases in *S. cerevisiae*, Kex2 and Prb1, localize to the trans-Golgi and the vacuole, respectively [12]–[14]. The localization of Kex2 and Prb1 to intracellular compartments raises the possibility that Osw3 may localize within the spore rather than at the spore wall. To determine Osw3 localization an *OSW3-GFP* fusion was constructed and introduced into wild-type cells. GFP fluorescence became visible about six hours after these cells were transferred to sporulation medium, when the bulk of the population had reached meiosis II (Figure 7B). Osw3-GFP could be seen initially in the ER in cells early meiosis and then in the prospore membrane as cells progressed into meiosis II. In mature spores, Osw3-GFP localized to the spore wall as well as the nuclear envelope/ER (Figure 7B). To confirm that Osw3-GFP is localized within the spore wall, Osw3-GFP localization was also examined in *dit1Δ* cells (Figure 7B). As with Spr1-GFP, Osw3-GFP diffused from the spore wall into the ascal cytoplasm of *dit1Δ* asci, demonstrating that Osw3-GFP is normally soluble in the periplasmic space within the spore wall.

### The presumptive active site of Osw3 is essential for pro-domain cleavage and function

**Figure 8:**
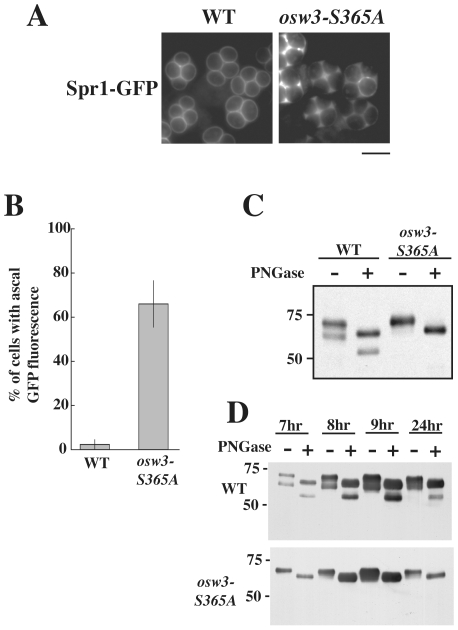
The Osw3 active site serine is essential for function. (A) YS478 (*OSW3*), and YS479 (*osw3-S365A*) were sporulated for 20 h and analyzed by fluorescence microscopy. Bar = 5 µm. (B) Distribution of Spr1-GFP in the strains in (A) was analyzed by fluorescence microscopy after 20 h. Data shown are the averages of three independent experiments. The vertical lines indicate the range of the values. (C) Western blot analysis of AN120 (wild type) carrying high copy plasmids expressing *OSW3-3HA* or *osw3-S365A-3HA* (pRS424- P_TEF2_-OSW3-3HA or pRS424- P_TEF2_-osw3-S365A-3HA). The tagged *OSW3* genes were expressed constitutively under the *TEF2* promoter and analyzed in vegetative cells. (D) Sporulation time course of AN120 (wild type) carrying high copy plasmids expressing *OSW3-3HA* or *osw3-S365A-3HA* under the *OSW3* promoter (pRS424-OSW3-3HA or pRS424-osw3-S365A-3HA). Samples were removed at the indicated times and examined with and without PNGase treatment. Subtilisin proteases are often expressed as zymogens that are activated by cleavage of the pro region [28]. Western blot analysis using anti-HA antibodies was used to examine the processing of Osw3-HA. *OSW3-HA* was expressed from the constitutive *TEF2* promoter and the mobility of the protein was analyzed in vegetative cells. Under these conditions the protein migrated as two distinct bands after SDS-PAGE fractionation (Figure 8C). To test whether these two bands were due to different glycosylated forms of the protein, extracts were treated with PNGase F to remove N-linked glycosylations before western analysis. After PNGase treatment, both the upper and lower Osw3-HA bands displayed increased mobility. This result demonstrates that both forms of Osw3-HA are glycosylated and that differential glycosylation does not create the two different mobilities.

To determine if protease activity of Osw3 is important for its function, an alignment with other subtilisins was used to identify the presumptive active site serine residue (S365) and this position was changed to alanine by site-directed mutagenesis. When *osw3^S365A^* was introduced into an *osw3Δ* strain, it failed to rescue the Spr1-GFP leakage phenotype (Figure 8A and B). This result suggests that Osw3 has protease activity and that this activity is essential for function.

Rather than distinct glycosylation states, the two bands may represent the pro- and active forms of the enzyme. Interestingly, when the S365A mutation was introduced into Osw3-HA and this protein was examined, only the slower migrating form of the protein was seen (Figure 8C). Taken with the complementation data, this result suggests that faster migrating, presumably activated, form of Osw3 is necessary for function and that Osw3 might activate itself by auto-cleavage of the pro-domain.

To ensure that these same processing events occur in sporulating cells, Osw3-HA and Osw3-S365A-HA, expressed from the native *OSW3* promoter, were examined by Western blot across a sporulation time course (Figure 8D). For the wild-type protein, both the processed and unprocessed forms were seen in cells progressing through meiosis (6 to 8 hour time points) and in mature spores (24 hour time point). For Osw3-S365, the protein was present but only the slower migrating form was seen. Thus, the failure of Osw3-S365 to be processed in vegetative cells is likely due to a defect in auto-cleavage and not to the absence of some other sporulation-specific processing factor.

## Discussion

The outer layers of the *S. cerevisiae* spore wall are necessary for spores' resistance to a variety of different environmental stresses. We show here that the outermost dityrosine layer creates a permeability barrier that restricts the diffusion of molecules, at least down to the size of GFP, out of the spore wall. Presumably, this barrier also limits access of exogenous proteins to the spore. Exclusion of proteins could account for the ability of the spore wall to resist digestion by glycanases as the permeability barrier created by the dityrosine layer would shield the inner carbohydrate layers from the digestive enzymes.

The physical structure of this barrier remains unknown. If one conceives of the β-glucan and chitosan layers as a meshwork of overlapping and cross-linked carbohydrate chains, then the diffusion of soluble molecules through the wall will be governed by the average pore size of that mesh. We report that the rate of diffusion of GFP out of the spore wall is similar in *chs3Δ* mutants, in which the GFP would have to diffuse out through only the β-glucan layer, and in *dit1Δ* mutants, in which both the β-glucan and chitosan layers are present. This suggests that the effective pore size of both these carbohydrate layers is similar and that the dityrosine layer is primarily responsible for creating the diffusion barrier of the spore wall.

The structure of the dityrosine polymer is not known, though a partially hydrolyzed preparation of the polymer contains molecules up to 100 kD in size [29]. This suggests that individual “chains” may have hundreds of dityrosine moieties. Whether these chains are linear or branched also remains to be determined. The dityrosine chains are likely cross-linked to the chitosan through the free amino group on the glucosamine [30]. Thus, one way to conceive of the barrier is as a branched polymer of dityrosine that is multiply cross-linked to different chitosan chains. By connecting the chitosan chains together, the dityrosine might physically occlude the pores in the chitosan mesh creating the diffusion barrier. Alternatively, dityrosine-mediated crosslinking might alter the geometry of the chitosan strands creating a smaller effective pore size.

Several of the permeability mutants found in this screen were also identified in an earlier screen for ether sensitivity (*gis1Δ*, *osw1Δ*, *osw2Δ*, *ydr326cΔ*, *far10Δ*) [10]. However, the correlation between sensitivity to volatile organics such as ether and permeability is not exact. Most of the permeability mutants identified here were not found in the earlier screen. Moreover, direct assay of *osw3Δ*, *osw4Δ*, and *osw5Δ* mutants indicates that they are resistant to ether treatment (R. K. R. and A. E. C., unpublished observations). This imperfect correlation suggests that the ether-resistance and leakage assays are interrogating different aspects of the spore wall structure. By contrast, all of the mutants with the strongest leakage phenotypes (*gis1Δ*, *osw1Δ*, *osw3Δ*, *osw4Δ*, *osw5Δ*) show increased sensitivity to treatment with β-glucanases (Figure 6 and [10]). The ability to resist enzyme treatment, in contrast to exposure to volatile organics, is a more direct consequence of the permeability of the spore wall.

Mutation of the *OSW3* gene caused increased spore wall permeability. *OSW3* encodes a subtilisin-related protease that is localized to the spore wall. Mutation of the presumptive active site serine in Osw3 causes both increased spore wall permeability and a failure of the protein to be processed from its pro-form. These results suggest that a protease activity of Osw3 is required for spore wall assembly and that the protein might remove its pro-region auto-catalytically. Members of the Kexin subfamily of subtilisin proteases often cleave at single or paired basic residues, while proteinase K family members generally have broader specificity [11], [28]. Alignment of Osw3 with orthologs from closely related yeasts revealed a conserved KLKK motif located between the pro-region and the protease domain near the presumed cleavage site. However, mutation of this motif alone, or in combination with other basic residues, did not impair the processing or the function of Osw3 (Y. S., unpublished observation). Consistent with the classification of Osw3 in the proteinase K subfamily [11], this observation suggests that Osw3 is not specific for cleavage after basic amino acids.

How the protease activity of Osw3 contributes to the diffusion barrier remains to be determined. The most straightforward possibility is that it is required for the processing and activity of one or more additional proteins necessary for proper assembly of the outer spore wall layers. The obvious candidates for such targets are the products of other genes involved in barrier formation. To test this possibility, the Osw1, Osw4, and Osw5 proteins were examined by Western blot in wild type and *osw3* cells, but no difference in mobility was seen (Y. S., unpublished observations). The presumptive protein target of Osw3 remains to be identified.

Another possibility is that Osw3 function is required not for processing of another protein but to act on the dityrosine layer itself. Analysis of the purified dityrosine polymer indicates that it lacks sugar constituents and is composed primarily of dityrosine as well as glycine and a smaller amount of other amino acids [27]. It could be that these components are connected through amide linkages as in peptide bonds. If so, these bonds may be subject to cleavage by a protease such as Osw3. Chitinases and glucanases play roles in the proper construction of the carbohydrate layers in both the vegetative cell wall and the spore wall [21], [31]-[33]. In an analogous fashion, Osw3 may be important for trimming the dityrosine chains to promote proper assembly.

In addition to *OSW3*, three of the four other mutants with the strongest leakage phenotypes (*osw1,4,5*) affect proteins that have been shown or are predicted to be localized to the spore wall compartment. *OSW3* has been previously identified in a screen for mutants altering RNA Polymerase I transcriptional activity and was named *RRT12* [34]. *OSW4* was also identified in an earlier screen as *LOH1*, a gene in which mutations cause an increased rate of loss of heterozygosity [35]. The connection between the spore wall phenotypes of *osw3* and *osw4* and these other roles is unclear. Micoarray experiments suggest that these genes are not expressed in vegetative cells but highly induced during sporulation [36], [37], though perhaps they are expressed at very low levels during vegetative growth. However, the sporulation-induced transcription of both genes, the localization of Osw3, and the predicted localization of Osw4 suggest that any effects of the mutants on nuclear events in vegetative cells are probably indirect. Rather, the Osw proteins are likely to be involved directly in assembly of the outer layers of the spore wall. The identification of these *OSW* genes is an important first step in understanding the machinery involved in synthesis of the outer spore wall layers.

The Dit1 and Dit2 enzymes involved in synthesis of the dityrosine precursor are conserved in a variety of yeasts, including pathogens such as *Coccidioides immitis* and *Candida albicans*. Given the importance of dityrosine in the spore wall for the resistance of spores to environmental stresses, it seems likely that dityrosine will play a similar role in these other fungi. Indeed, mutation of the *C. albicans* ortholog of *DIT2* has been shown to cause cell wall defects [38]. A better understanding of the dityrosine assembly pathway in *S. cerevisiae* may, therefore, provide insight into the construction of cell walls in these human pathogens.
